# TDRD3 is an antiviral restriction factor that promotes IFN signaling with G3BP1

**DOI:** 10.1371/journal.ppat.1010249

**Published:** 2022-01-27

**Authors:** Matthew Deater, Manasi Tamhankar, Richard E. Lloyd

**Affiliations:** Department of Molecular Virology and Microbiology, Baylor College of Medicine, Houston, Texas, United States of America; Stanford University, UNITED STATES

## Abstract

Stress granules (SGs) are highly dynamic cytoplasmic foci that form in response to activation of the integrated stress response (ISR) that results in eIF2α phosphorylation and global translation shutdown. Stress granules, which are largely nucleated by G3BP1, serve as hubs for mRNA triage, but there is mounting evidence that they also perform cell signaling functions that are vital to cell survival, particularly during viral infection. We previously showed that SG formation leads to NFκB activation and JNK signaling and that this association may be due in part to G3BP1-dependent recruitment of PKR to SGs. Others have reported close associations between G3BP1 and various innate immune PRRs of the type 1 interferon signaling system, including RIG-I. We also reported SG assembly dynamics is dependent on the arginine-methylation status of G3BP1. Another protein that rapidly localizes to SGs, TDRD3, is a methyl reader protein that performs transcriptional activation and adaptor functions within the nucleus, but neither the mechanism nor its function in SGs is clear. Here, we present evidence that TDRD3 localizes to SGs partly based upon methylation potential of G3BP1. We also characterize granules that TDRD3 forms during overexpression and show that these granules can form in the absence of G3BP but also contain translation components found in canonical SGs. We also show for the first time that SGs recruit additional interferon effectors IRF3, IRF7, TBK1, and Sting, and provide evidence that TDRD3 may play a role in recruitment of these factors. We also present evidence that TDRD3 is a novel antiviral protein that is cleaved by enteroviral 2A proteinase. G3BP1 and TDRD3 knockdown in cells results in altered transcriptional regulation of numerous IFN effectors in complex modulatory patterns that are distinctive for G3BP1 and TDRD3. Overall, we describe a novel role of TDRD3 in innate immunity in which G3BP1 and TDRD3 may coordinate to play important roles in regulation of innate antiviral defenses.

## Author summary

When cells are exposed to environmental stresses, such as oxidative stress and viral infection, it induces a cellular response leading to the formation of Stress Granules (SGs) composed of stalled translation initiation complexes (RNA-binding proteins and mRNA) and many other cellular proteins. SGs are also considered to be antiviral structures when they form during viral infection, but viruses can block SG formation to facilitate their survival, often by targeting the essential SG protein G3BP1. Here, we show that a methyl reader protein, TDRD3, localizes to SGs partly based on the methylation potential of G3BP1, and may play a role in the recruitment of innate immune factors to SGs. Further, when overexpressed, TDRD3 can also form SG-like structures independently of G3BP1. We also present evidence that TDRD3 is a novel antiviral protein. Virus replication is enhanced in the absence of both TDRD3 and G3BP1, and virus infection leads to cleavage of TDRD3 by the enterovirus proteinase 2A. Finally, we also show that depletion of TDRD3 and G3BP1 together in cells leads to restriction of transcriptional activation of numerous IFN effectors in response to dsRNA. The patterns of transcriptional activation are distinctive for G3BP1 and TDRD3. We conclude that TDRD3 may play a novel and important role in the regulation of the host antiviral response.

## Introduction

Stress granules (SGs) are messenger ribonucleoprotein (mRNP) condensates, consisting largely of stalled translation initiation complexes of mRNAs which form in response to various homeostatic insults [1,2]. For instance, upon detection of viral dsRNA intermediates, the cytosolic dsRNA sensor PKR phosphorylates eIF2α leading to eIF2α-dependent translation shutoff. When untranslated mRNA increases within the cytoplasm, the major nucleator of SG formation, G3BP1, assembles stalled translation initiation complexes with other RNA binding proteins (RBPs) through liquid phase transitions into stress granules [3]. SG assembly is a complex process involving multiple RNA-RNA, RNA-protein, and protein-protein interactions [4,5]. Recent evidence also suggests this process is selective, wherein constitutively expressed mRNAs remain silenced via compartmentalization, while allowing translation of select other mRNAs with protective reparative functions [6,7].

While there are four known eIF2α kinases (GCN2, PERK, HRI and PKR) that are activated during different types of environmental stresses, their downstream signaling ultimately converges upon stress granules [1]. This integration of signaling via divergent kinases resulting in a concerted response is referred to as the integrated stress response (ISR) [8]. A key player in ISR, G3BP1, is becoming increasingly known for its role in promoting innate immune antiviral responses. An association between SGs and some type 1 interferon signaling effectors has been reported, with recruitment of antiviral Pattern Recognition Receptor (PRR) effectors OAS, RIG-I, MDA5, RNase L, PKR to SGs [9,10]. We previously showed that NFkB signaling increases with an increase in G3BP1-induced SG formation and recruitment of PKR by G3BP1 leads to its activation [11]. Apart from colocalization into SGs however, the exact function of recruitment of various PRR sensors into SG remains unclear. In addition to PKR activation through SG recruitment, G3BP1 by itself has been shown to promote DNA binding and activation of the DNA sensor cGAS, leading to the subsequent activation of type 1 interferon signaling [12]. G3BP1 is also associated with and promotes the expression of RIG-I [13]. G3BP1 also binds HCV viral dsRNA leading to the upregulation of interferon beta expression. This was mediated by G3BP1’s arginine-rich RGG domain [14]–a disordered region at the C-terminus of G3BP1 that is a site for arginine methylation [15].

Previously, we showed that dynamic methylation and demethylation of G3BP1’s RGG domain facilitates SG formation kinetics, and these methylation events are catalyzed by the protein arginine methyl transferases (PRMTs) 1 and 5 [15]. PRMT1 mediated asymmetric methylation plays a key role in SG formation kinetics while PRMT5 mediated symmetric methylation may play an as yet unknown regulatory role in SG biology [15,16]. TDRD3, which is a largely nuclear protein with methylarginine-reading and transcriptional activation adaptor functions [17,18] has been shown to rapidly localize to SGs via its Tudor domain [19]. However, the mechanism for localization to SGs and its potential functions within SGs remain unknown. TDRD3 itself has been shown to form granule-like foci within the cytoplasm upon overexpression [20], but details of these foci have not been reported. Considering that SG assembly kinetics are governed by dynamic methylation of arginine residues within G3BP1’s RGG domain, and that TDRD3 binds these methyl-arginine marks of other proteins [21], we investigated if TDRD3 is recruited to SGs based upon methylation of G3BP1 and provides any adaptor functions related to antiviral responses. Here, we present evidence that RGG domain methylation plays a limited role in TDRD3 recruitment to SGs, that TDRD3 is involved in recruitment of IFN effectors IRF3, IRF7 and TRAF3 to SGs, and that TDRD3 is a novel antiviral restriction factor. Further we show that TDRD3 and G3BP1 co-modulate IFN effector and inflammatory signaling transcriptional responses in response to dsRNA in cells.

## Results

### G3BP1-induced SGs recruit type 1 interferon effectors

**Fig 1:**
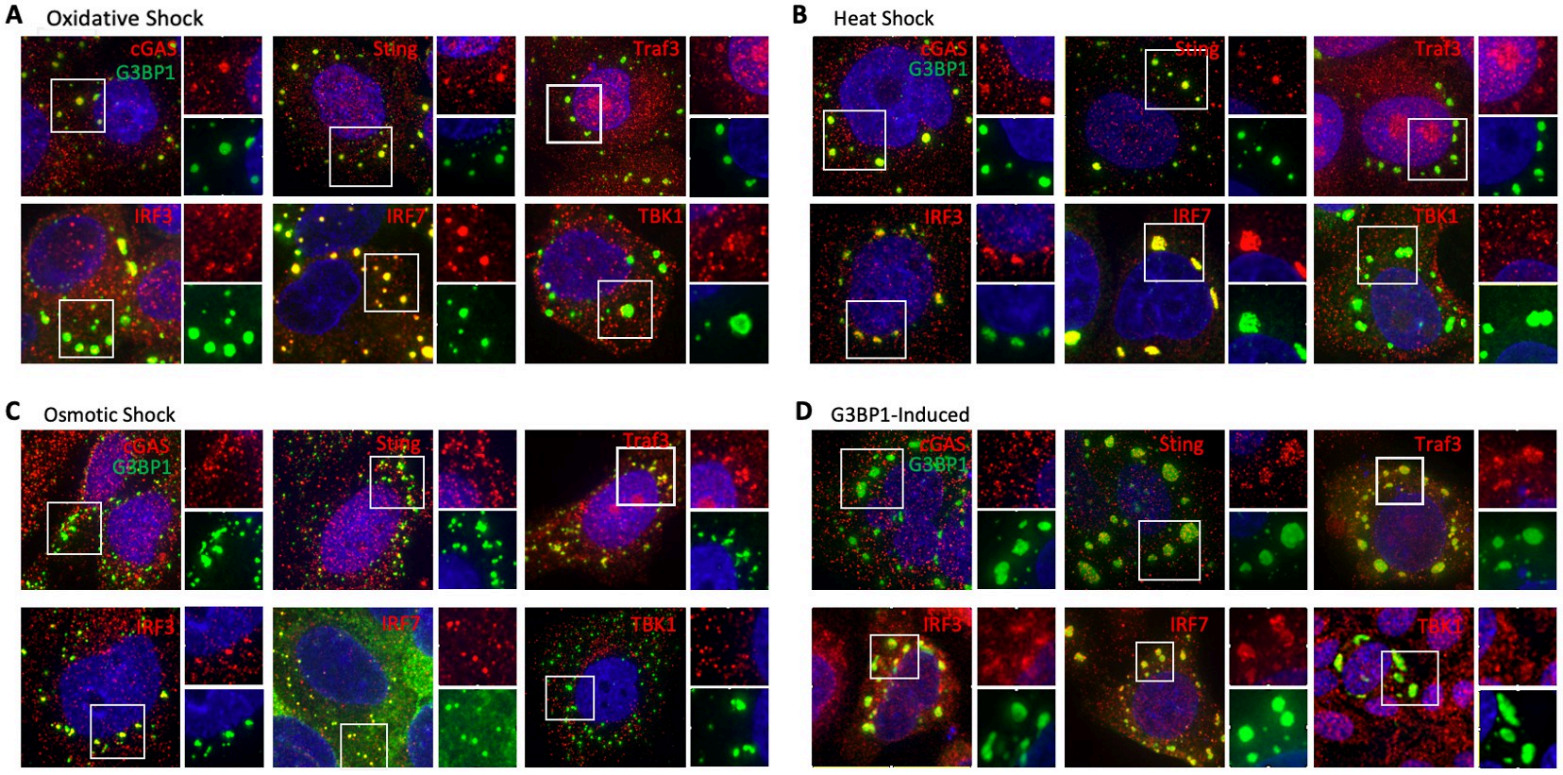
IFN-I effectors are recruited to G3BP1-induced and various stress-induced SGs. (A-C) U20S cells were treated to produce oxidative shock, heat shock or osmotic shock SGs as described in the Materials and Methods, then fixed and stained for IRF3, IRF7, TBK1, TRAF3, Sting or cGAS. IFA was then performed using deconvolution microscopy. (D) G3BP1-GFP was transiently expressed in U20S cells for 18 hrs to allow formation of SGs, which were then subjected to IFA microscopy for innate factors indicated as described in the Materials and Methods.

Previous studies have shown that the cytosolic dsRNA sensors MDA5, RIG-I, and PKR localize to SGs [9,10,14]. However, the role of signaling kinases and upstream activators within the type 1 interferon signaling pathway have remained unexplored, thus the mechanism(s) of how SGs function as signaling platforms is unclear. To further examine how SGs serve as signaling platforms, we treated U20S cells with various stressors that produce SGs with different assembly pathways [1], and then examined granules for recruitment of additional key components of the IFN signaling pathway, type 1 interferon regulatory factors (IRFs) 3 and 7, as well as their direct upstream kinase TBK1 and the receptor-associated signaling initiator TRAF3. We also examined Sting and cGAS for incorporation since only cGAS has been reported to interact with G3BP1 [22]. We found that stress granules induced by oxidative stress (arsenite) strongly recruited IRF7 and more weakly recruited IRF3, TRAF3 and TBK1, STING and also recruited cGAS as reported [22] (Fig 1A). Heat shock induced SGs also recruited IRF7, IRF3, TRAF3, as well as STING and cGAS, but not TBK1 (Fig 1B). SGs induced by osmotic shock were smaller but also strongly recruited IRF7, IRF3 and TRAF3, weakly recruited Sting, but did not recruit TBK1 or cGAS (Fig 1C). Stress granules can also be induced in the absence of exogenous stressors by overexpression of the dominant SG nucleating protein G3BP1 in G3BP-null U20S cells. Fig 1D shows that IRF3, IRF7, TRAF3 and TBK1 colocalized to these SGs, STING was weakly recruited and cGAS was not recruited. Although G3BP1 is found in >90% of stress granules formed by these three pathways, oxidative stress activates PKR through HRI, heat shock only weakly induces eIF2a phosphorylation and osmotic shock activation of SG occurs through phospho-eIF2α-independent mechanisms [1], which may account for differential localization of some IFN effectors.

### G3BP1 RGG methylation plays a limited role in TDRD3 recruitment to SG

**Fig 2:**
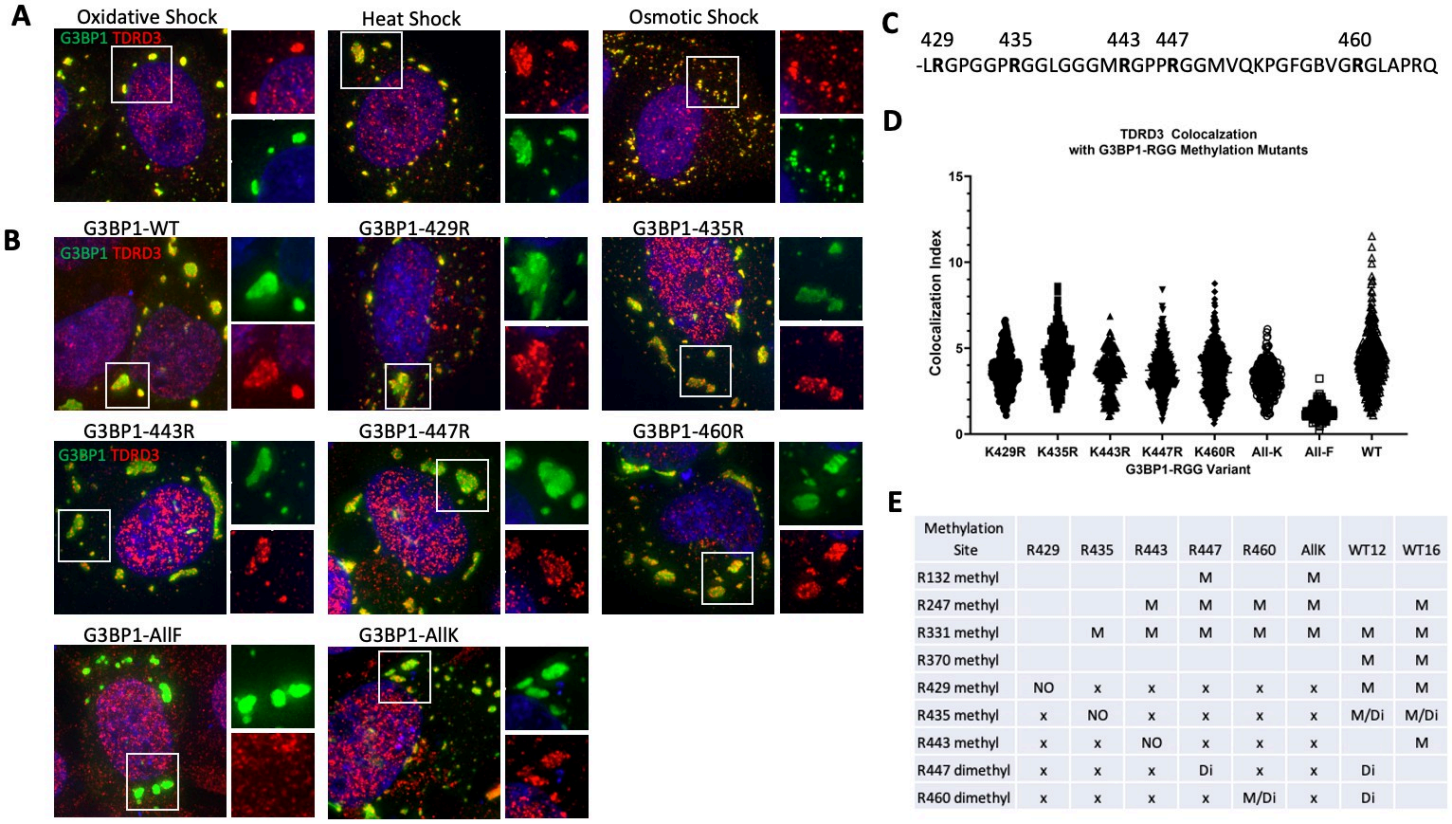
TDRD3 is recruited to different types of SGs and G3BP1 mutants with certain RGG arginine residues. (A) WT U2OS cells were treated with indicated stressors to induce types of SG and stained for IFA analysis for G3BP1 (green) or TDRD3 (red). (B) G3BP-null U2OS cells were transfected with wild type (WT) G3BP1-GFP, G3BP1 with R to F mutations on key arginines (All-F), G3BP1 with R to K mutations on key arginines (All-K), or variants containing single K-R point mutations on the All-K background at previously investigated arginine residues within the RGG domain. Colocalization of TDRD3 was determined by IFA analysis using anti-TDRD3 antibody (red). Deconvolution microscopy was performed and colocalization indices were calculated using Cell Profiler. Vignettes show examples of TDRD3 colocalization to each G3BP1 variant. (C) Sequence of the RGG domain of G3BP1 with denoted arginine residues that were mutated. (D) TDRD3 colocalizes to G3BP1 single arginine variants differentially. Results were plotted using Graphpad Prism. E. Mass spectroscopic analysis results of methylation on immunoprecipitated G3BP1 WT and RGG methylation mutants. M = monomethylated, Di = dimethylated, x = no observed methylation, NO = methylation was not observed where expected.

G3BP1 is not known to directly associate with the IFN signaling effectors studied here, leading to the possibility that other adapter proteins may exist that are potentially working with G3BP1 to facilitate recruitment of signaling effectors to SGs. TDRD3 was previously reported to enter arsenite-induced SGs but no function has emerged for TDRD3 in the context of stress granules [19]. To examine if TDRD3 is recruited to other types of SGs that assemble through other pathways, we treated U20S cells with osmotic shock or heat shock and found that TDRD3 was recruited to SGs under these conditions, as well as during oxidative stress as reported [19] (Fig 2A).

The mechanism of recruitment of TDRD3 to SGs during cell shock or G3BP1-overexpression is unclear. We hypothesized that TDRD3, which is a known methyl-arginine reader, localizes to SGs based on differential G3BP1-RGG methylation or that G3BP1-RGG methylation may stabilize TDRD3’s localization to SGs. To test this hypothesis, we created a series of G3BP1 variants wherein all previously determined key arginine residues within the RGG domain [15] (Fig 2C) were mutated to lysine except for one. Lysine cannot be methylated by PRMTs, thus this approach allows for only one potential methylation target for PRMT in the RGG domain of each G3BP1 variant. We transfected G3BP-null U2OS cells with these GFP-tagged G3BP1 RGG variants and assayed for the presence of TDRD3 in the resulting granules. (Fig 2B) shows that TDRD3 colocalizes to all single arginine G3BP1 RGG-variants tested but not the All-F variant. We quantified the degree to which TDRD3 colocalizes to these foci with G3BP1 RGG single arginine variants using a customized Cell Profiler analysis pipeline. Based on the results (Fig 2D), it appears that TDRD3 is recruited to G3BP1-induced SGs based on the remaining arginine within the RGG domain with only minor variations. Conservation of arginines at 435 and 447 allowed stronger TDRD3 recruitment, similar to WT G3BP1. Interestingly, there is also colocalization to granules produced with the G3BP1 variant wherein all key arginines of the RGG domain were reverted to lysine (All-K), indicating that methylation within the RGG domain is not required.

To confirm that methylation was actually occurring where expected on these G3BP1 methylation site mutants, each G3BP1 construct was expressed and purified for mass spectroscopic analysis to determine actual use of methylation sites on the constructs. Fig 2E shows summarized results. The mutants in the RGG domain G3BP1-K447R and G3BP1-K460R did indeed appear to be methylated where intended, while G3BP1-All-K prevented all methylation within the RGG domain as expected. Conversely, G3BP1-K429R, -K435R, and -K443R were not methylated at the indicated sites, suggesting methylation at these sites may occur secondary to methylation at another site in the RGG. Interestingly, we did detect methylation at previously unreported arginines R247 and R331, but the significance of methylation at these residues remains unknown, and individual methylation marks do not correspond with TDRD3 colocalization. We also included WT G3BP1 overexpressed for 12 hours and 16 hours to evaluate potential kinetic differences in methylation upon overexpression as we previously assessed arsenite stress granule formation and G3BP1 RGG methylation to be a highly dynamic process involving patterns of methylation leading to differences in SG formation kinetics [15]. Indeed R447-R460 dimethylation appeared at 12 hours but was not detected at 16 hours, and R247 monomethylation emerged at 16 hours showing kinetic differences in G3BP1 RGG methylation. Together, the results indicate that TDRD3 is not likely recruited to SG based on RGG methylation marks, though other methylation sites in G3BP1 may play roles or other factors in co-complexes with G3BP1 mutants.

### TDRD3 overexpression induces SG-like foci in the absence of G3BP1 and G3BP2

**Fig 3:**
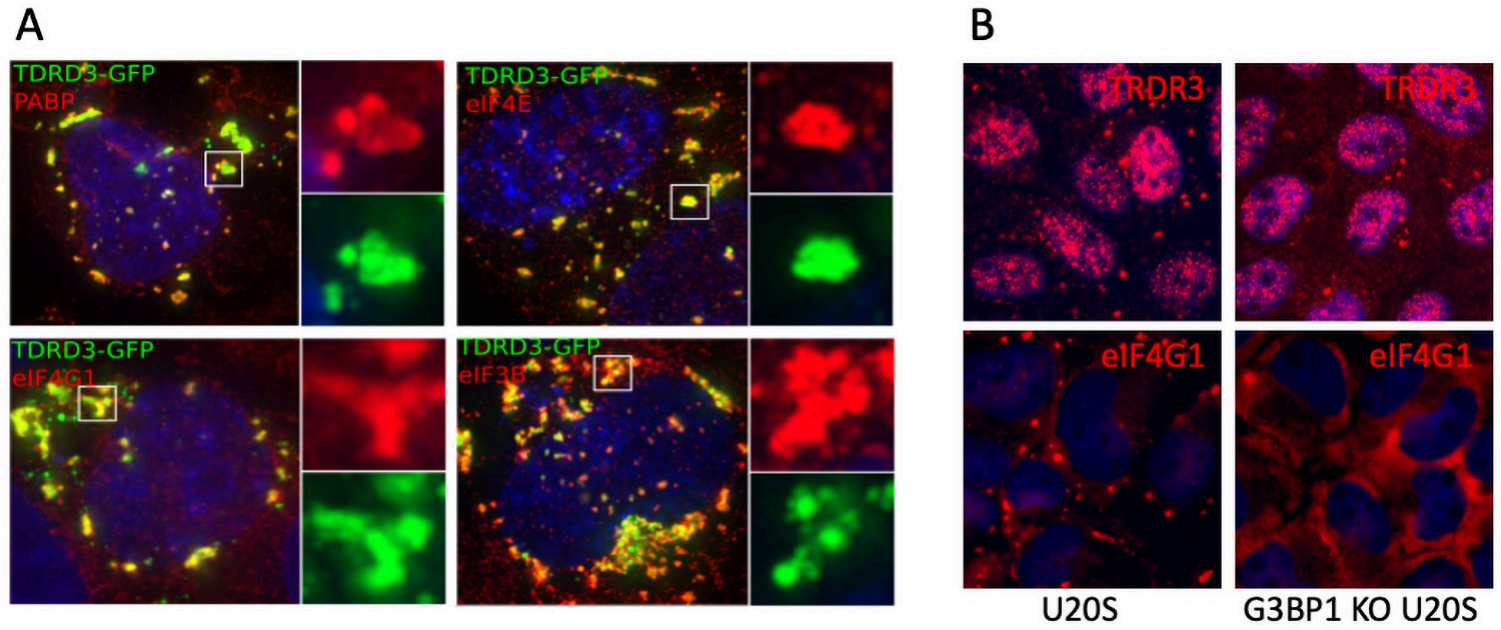
Overexpression of TDRD3-GFP induces cytoplasmic foci that contain canonical stress granule components. (A) G3BP-null U2OS cells were transfected with TDRD3-GFP expression plasmid for 18 hrs then processed for IFA with antibodies against the indicated translation initiation factors. (B) WT U20S cells or G3BP-null U20S cells were stressed with arsenite for 60 min and stained for the stress granule marker eIF4GI or TDRD3.

Previous studies [19] reported that TDRD3 overexpression can form granules within the cell that are G3BP1 and Tia1 positive much like SGs that form under stress or when G3BP1 is overexpressed. Since G3BP1 is a dominant nucleator of SG formation, but the role of TDRD3 in SG biology is unknown, we wanted to determine if TDRD3 overexpression in the absence of G3BP1 and G3BP2 could also trigger granule formation. To ascertain this, we overexpressed TDRD3-GFP in cells lacking G3BP1 or G3BP2 and found that granules do form in this cell type (Fig 3A). We then sought to determine if these granules–which share morphological characteristics with canonical and G3BP1-induced SGs, also contain canonical SG components consistent with incorporation of stalled translation pre-initiation complexes [8]. While not exhaustive, TDRD3-induced granules were found to recruit poly-A binding protein (PABP) and translation initiation factors eIF3B, eIF4E, and eIF4G1, similar to canonical stress granules (Fig 3A). Following this, we wanted to see if endogenous TDRD3 positive foci would form in the same G3BP-null cells under conditions of cell stress. After application of oxidative stress (ARS) in G3BP-null cells, the loss of which strongly hinders SG formation upon arsenite stress [23], we observed a few foci containing TDRD3, but lacking the canonical SG marker eIF4GI (Fig 3B). Since SGs form as a result of translation repression, we performed ribopuromycylation assays to determine if TDRD3 overexpression results in translation inhibition both in the presence or absence of G3BP (S1C Fig). These results suggest that neither G3BP1 nor G3BP2 are absolutely required for the condensation of TDRD3 into SGs, and in case of TDRD3 induced SG-like foci, the recruitment of the canonical stress granule components assayed were not dependent upon G3BP1 or G3BP2 and that TDRD3 overexpression will also lead to host translation shutoff.

### Type-I interferon effectors localize to TDRD3-induced foci

**Fig 4:**
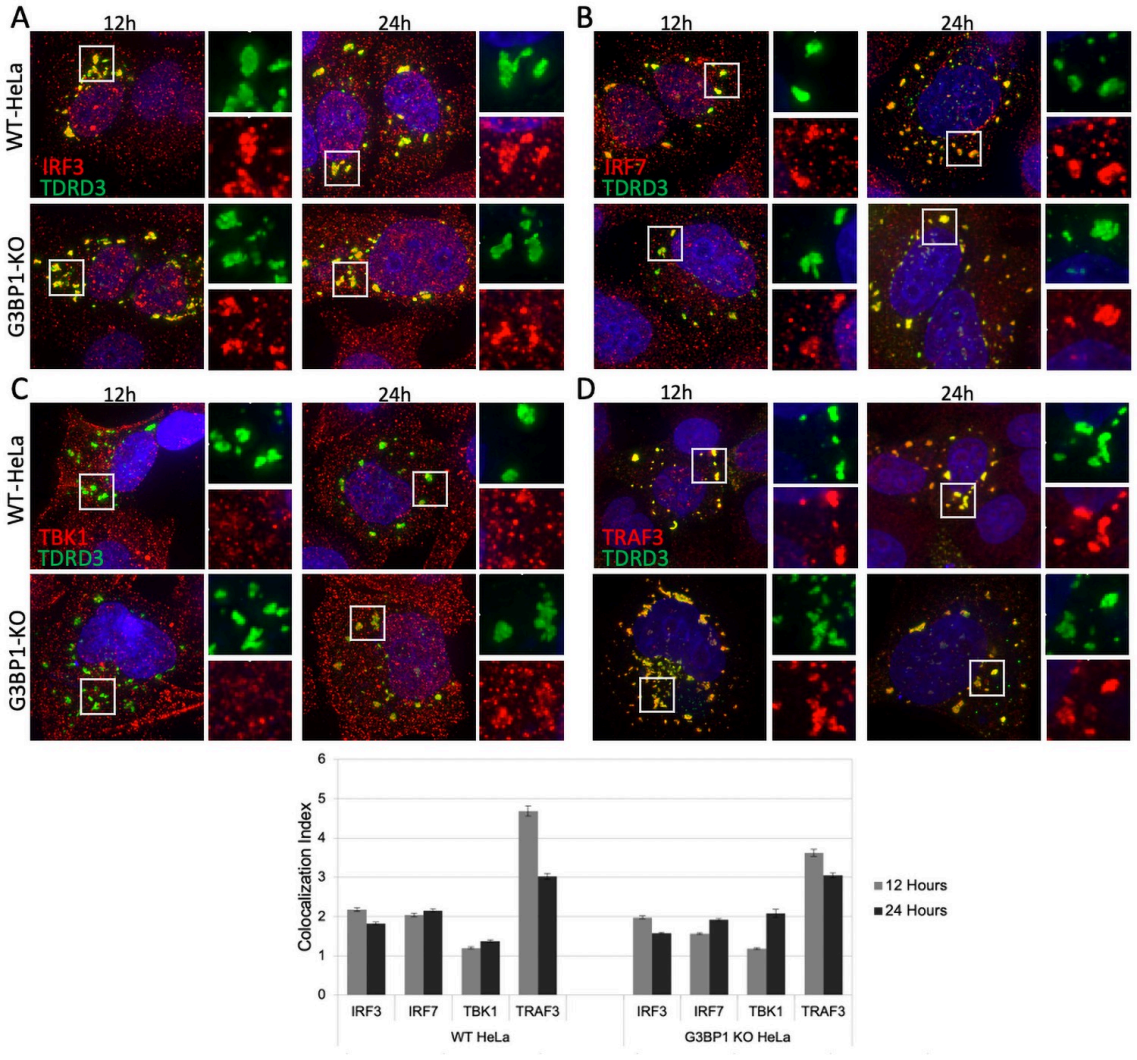
Interferon effectors localize to TDRD3-induced SG-like foci with or without G3BP1. G3BP1-KO or WT HeLa cells were transfected with GFP-tagged TDRD3 and incubated for 12 or 24 hours. Cells were then fixed and stained for: (A) IRF3, (B) IRF7, (C) TBK1, (D) TRAF3. (E) Resulting IFA image quantification by Cell Profiler.

Considering that G3BP1-induced SGs recruit type-1 interferon effectors (Fig 1), that G3BP1 plays a role in recruitment of TDRD3 to these SGs (Fig 2), and that TDRD3 itself can form SG-like foci even in the absence of G3BP1 (Fig 3), we wanted to see if TDRD3 by itself could recruit IFN-I effectors. To address this, we overexpressed TDRD3 in WT and G3BP-KO cells for 12 and 24 hours and then stained for the same IFN-I effectors that localized to G3BP1-induced SGs (Fig 4). We found that TDRD3 expression does recruit the type 1 interferon effectors IRF3, IRF7, TBK1, and TRAF3 to SG-like foci with or without G3BP1 present. When quantified, TDRD3 more strongly recruited TBK1 to SG-like foci at 24 hours of expression compared to cells expressing endogenous G3BP1, but overall the recruitment to TDRD3-induced granules occurs but is dampened in G3BP1 knockout cells. To explore this further, we treated WT, TDRD3 KD, G3BP1 KO, or KD/KO cells with arsenite and assayed for both Tia1-positive granule formation as a measure of stress granule formation, as well as these granules’ ability to recruit TRAF3 (S1D Fig). We found that in TDRD3 knockdown cells, granules still form, but recruitment of TRAF3 to these granules is greatly hindered–similar to what is seen in G3BP1 KO cells, compared to WT cells, while KD/KO cells show reduced granule formation as expected with no recruitment of TRAF3. Overall these results imply that G3BP1 and TDRD3 may be working in concert to recruit these type 1 interferon effectors.

### TDRD3 is recruited to virus-induced granules along with TRAF3 and IRF7

**Fig 5:**
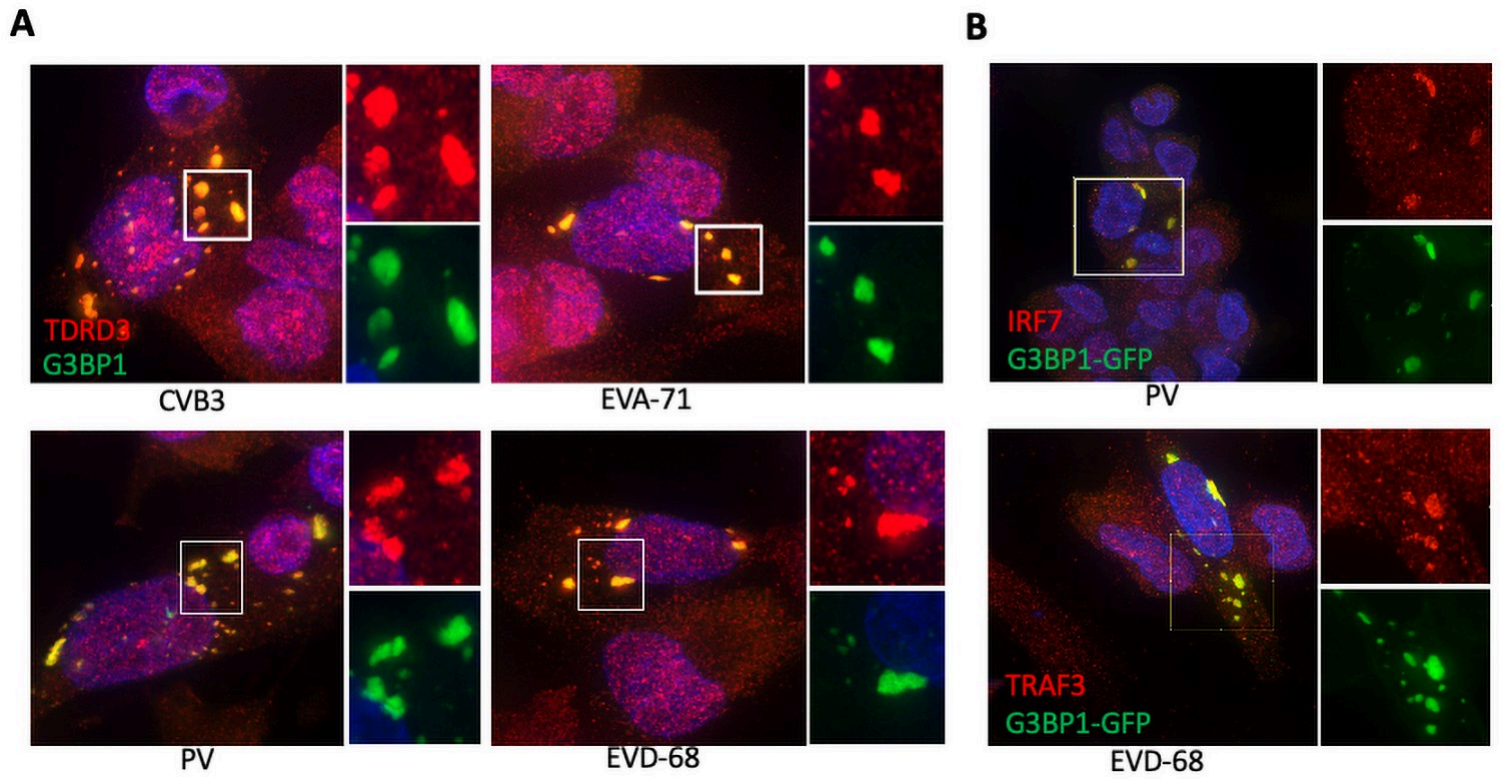
TDRD3 is recruited to virus-induced stress granules. (A) HeLa cells were infected (MOI = 1) for 6–16 hr with indicated enteroviruses before fixation and processing for IFA with antibodies to G3BP1 and TDRD3. (B) Cells infected with PV were stained for IRF7 and G3BP1 or cells infected with EVD-68 were stained for TRAF3 and G3BP1.

To examine TDRD3 in the context of virus infection, we next sought to determine if TDRD3 itself is recruited to virus-induced SGs. To address this, we infected HeLa cells with virus serotypes from four different enterovirus species, A, B, C, and D (*enterovirus A-71*, *coxsackievirus B-3*, *poliovirus*, *and enterovirus D-68*). After 5 hrs cells were fixed and stained for TDRD3 and G3BP1. Fig 5A confirms that TDRD3 does indeed localize to enterovirus-induced SGs. Further, we examined if type 1 interferon signaling effectors also localize to these virus-induced granules. Fig 5B confirms that both TRAF3 and IRF7 also localize to enterovirus-induced granules.

### Enteroviral 2A proteinase facilitates the cleavage of TDRD3

**Fig 6:**
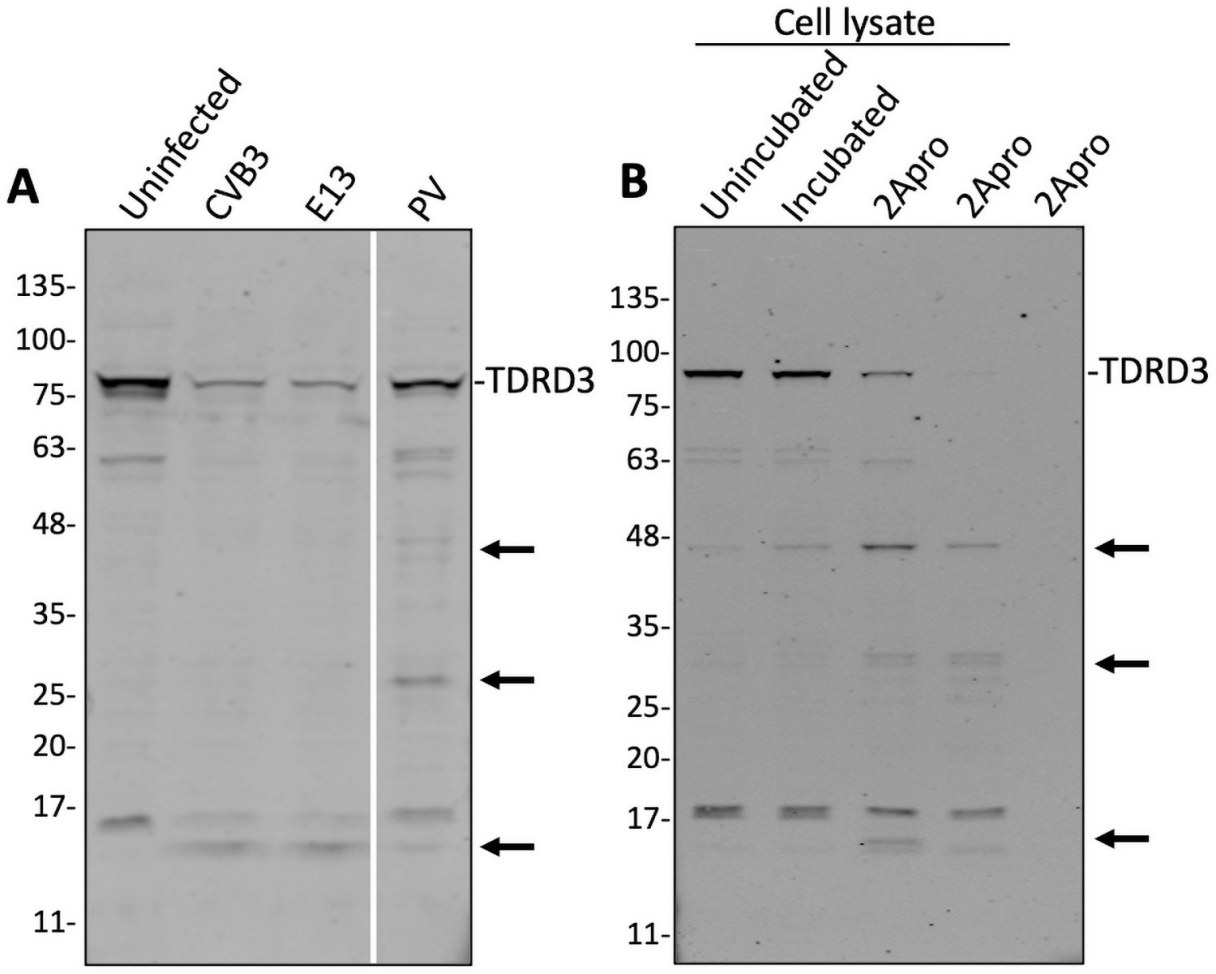
Enteroviral proteinase cleavage of TDRD3. (A) WT HEK cells were infected (MOI = 10) with coxsackie virus B3, echovirus 13, and poliovirus and incubated for 16 hours. Cells were then lysed and total protein concentration was determined via spectrophotometry. 100 μg total protein was loaded into each well and PAGE/western blot against TDRD3 was performed. Novel cleavage products were observed in infected cell lysates (arrows). (B) 100 μg of HEK WT whole cell lysate was either frozen (T = 0) or incubated with either glycerol (No Pro) or 5μg (first 2Apro) or 10 μg (second 2Apro) of 2A proteinase overnight at 37°C. PAGE was performed using 100 μg (total) WCL per lane. Western blot was performed against TDRD3.

We hypothesized that if TDRD3 indeed plays a significant role in antiviral immunity during enteroviral infection, then enteroviruses may have evolved to counteract TDRD3 function. Enteroviral 2A proteinase (2A^pro^) or 3C proteinase (3C^pro^) are known to cleave many critical cellular targets to enable viral sequestration of host machinery and processes. These viral proteinases also cleave multiple IFN signaling factors, including RIG-I, MDA-5, MAVS [24–27] and G3BP1, which also has antiviral function [28,29]. To determine if TDRD3 is also targeted by enteroviral proteinases, we infected HEK cells with coxsackievirus B3, echovirus 13, or poliovirus for 8 hours and performed western blot analysis of TDRD3. Fig 6A shows that TDRD3 is unstable and reduced in cells during infections with these enteroviruses. Further, a number of new smaller TDRD3-specific proteins are produced, likely cleavage products. To determine which viral proteinase was involved in TDRD3 cleavage, HEK cell lysates were incubated alone or with purified recombinant CVB3 2A^pro^ which resulted in a decrease or abrogation of intact TDRD3 and similar cleavage products noted during virus infection. This indicates that 2A proteinase causes proteolytic processing of TDRD3, either directly or indirectly.

### G3BP1 and TDRD3 together limit enteroviral replication

**Fig 7:**
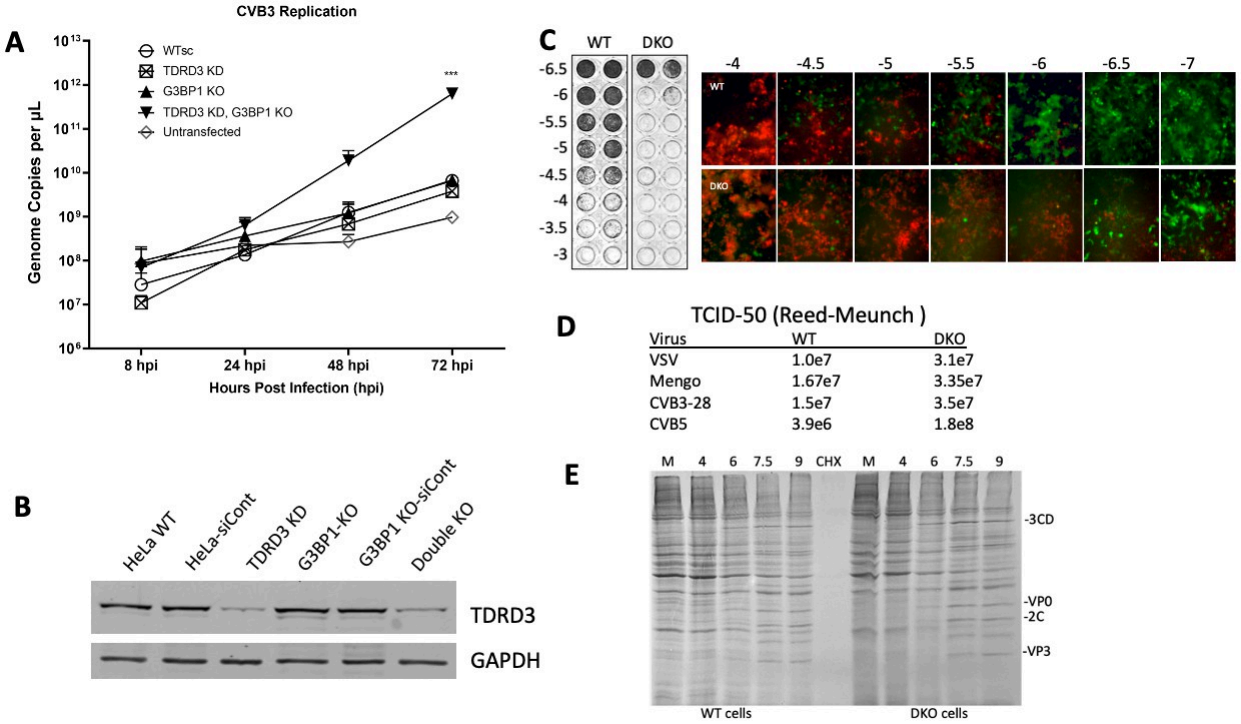
G3BP1 and TDRD3 together restrict enteroviral replication. WT HeLa cells possessing scrambled shRNA (WTsc) or G3BP1 knockout HeLa cells possessing scrambled shRNA (G3BP1 KO), TDRD3 knockdown cells, or cells with both G3BP1-KO and TDRD3-KD were infected with CVB3 (MOI = 0.5) and incubated for up to 72 hours. (A) Supernatants were retained for each timepoint and analyzed via RT-qPCR for absolute quantification of genome copies. Statistics: Significance was determined using two-way RM ANOVA; P value < 0.0001. (B) Western blot showing efficiency of TDRD3 silencing in TDRD3 knockdown cell lines. (C) Infectivity assays with indicated serial dilutions of virus with crystal violet endpoint stain or microscopy using dsRED-CVB3 were carried out in WTsc HeLa cells and cells with both G3BP1-KO and TDRD3-KD for 48 hrs. Both cell types express GFP. (D) Reed-Meunch TD50 infectivity assays were carried out with VSV, Mengo, CVB3-28 or CVB5 in the same two cell types. (E) Single step growth curves with MOI = 3 of CVB3-28 showing translation in cells at indicated timepoints were pulse labeled with 35S-methionine/cysteine. Autoradiograph of SDS-PAGE analysis of radiolabeled cell lysates are shown.

To further test the hypothesis that TDRD3 may play an antiviral role during infection, particularly in the context of SGs and G3BP1, we infected HeLa cells with dsRed-expressing CVB3 at an MOI of 0.5 to generate multistep viral growth curves. HeLa cells infected were either WT, G3BP1-KO, TDRD3 knockdown (KD), or cells with both G3BP1-KO and TDRD3 KD (KD/KO). In cells lacking G3BP1, viral replication was increased modestly as expected, but only through 24 hpi (Fig 7A). Surprisingly, TDRD3-KD cells appeared to slightly restrict viral replication compared to non-silencing controls. However, when both G3BP1 and TDRD3 were absent or reduced, viral replication increased to much higher levels by 48 or 72 hpi. We confirmed this observation by performing TCID infectivity assays in WT and KD/KO HeLa cells (Fig 7C) which revealed much higher infectivity of CVB3 in KD/KO cells. This was also confirmed with TD50 assays with enteroviruses. Similar, but weaker effects were noted with VSV and mengovirus (Fig 7D). Furthermore, we performed high MOI single step growth curves of CVB3 followed by radioactive pulse labeling of protein synthesis (Fig 7E) which revealed no discernable differences in viral translation, host translation shutoff or virus polyprotein processing in KD/KO cells compared to WT HeLa cells. Overall, this indicates that G3BP1 and TDRD3 function together in antiviral roles, primarily at the level of virus infectivity.

### TDRD3 and G3BP1 together modulate transcriptional activation of interferons, IRFs, ISGs, and pro-inflammatory cytokines

Considering that KD/KO cells have enhanced virus infectivity, and that IFN signaling effectors localize to canonical as well as G3BP1- and TDRD3-induced foci, we next wanted to determine whether Type 1 Interferon signaling is impaired in KD/KO cells. To address this, we transfected these cells with poly I:C concentrations ranging from 0 to 1 ug/ml. Additionally, we infected cells with CVB3 expressing dsRED at an MOI of 0.5 and incubated cells at indicated times ranging from 0 to 48 hours post infection. We then performed RT-qPCR analysis of expression of IFN-I signaling ligands (interferons α, β, λ1, and λ2/3) as well as transcriptional activators (IRF1, IRF3, IRF7, and IRF9) and various effectors and other ISGs involved in antiviral immunity (ISG15, ISG20, ISG56, Viperin, IFI6, ZAP, OAS, ADAR1, RTP4, RNaseL, SOCS1, and SOCS3) as well as pro-inflammatory cytokines (CXCL9, CXCL10, CXCL11, and IL-1β).

**Fig 8:**
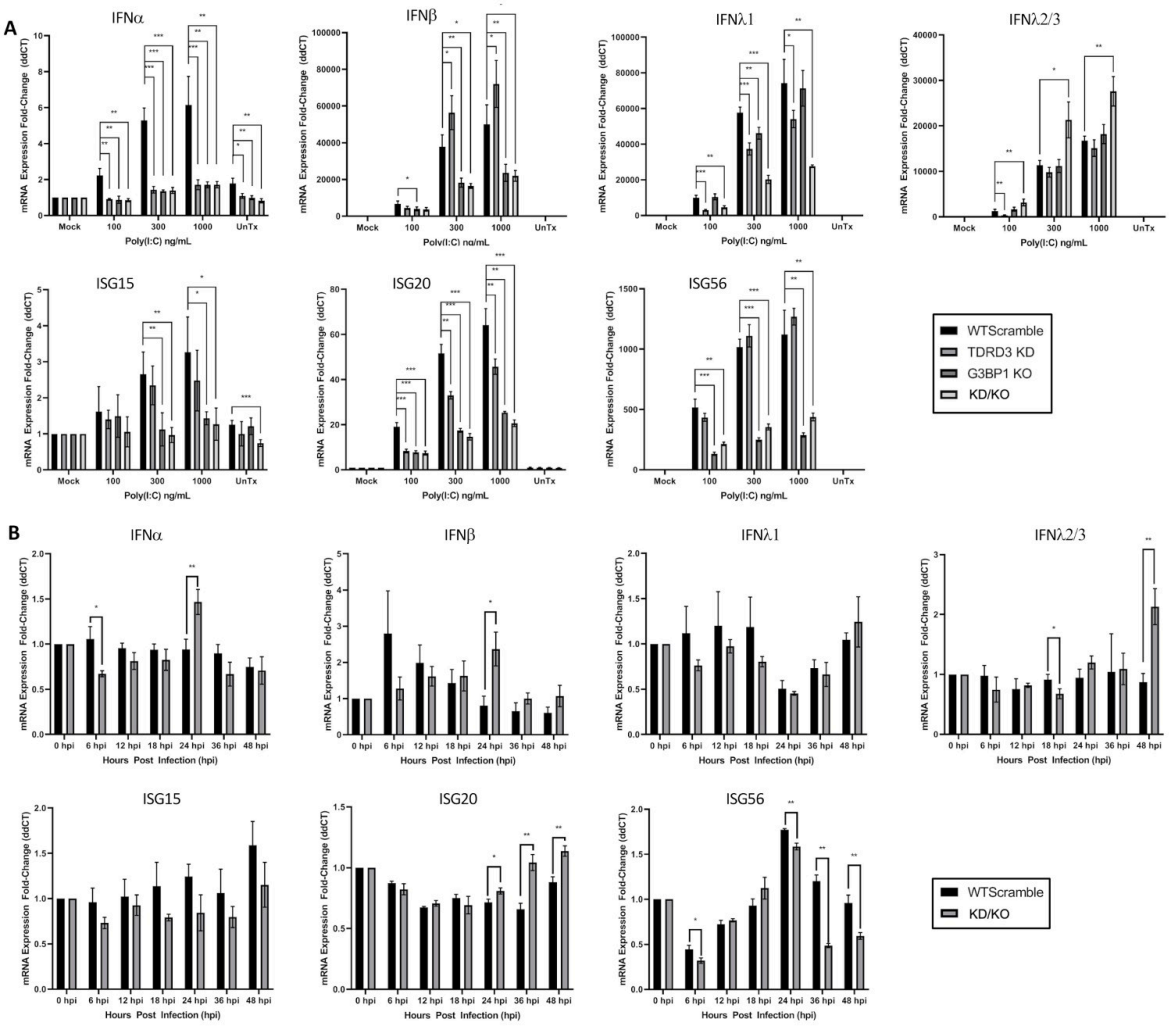
TDRD3 and G3BP1 together modulate transcriptional activation of Types 1 and 3 interferons and ISGs. (A) WT HeLa cells possessing scrambled shRNA (WTsc) or G3BP1 knockout HeLa cells possessing scrambled shRNA (G3BP1 KO), TDRD3 knockdown cells, or cells with both G3BP1-KO and TDRD3-KD were transfected with low molecular weight poly(I:C) at concentrations of 0 ng/mL (Mock), 100 ng/mL, 300 ng/mL, 1 μg/mL, or untransfected (UnTx). Cells were incubated for 12 hours post transfection. (B) WT HeLa cells possessing scrambled shRNA (WTsc) or G3BP1 knockout HeLa cells possessing scrambled shRNA (G3BP1 KO), TDRD3 knockdown cells, or cells with both G3BP1-KO and TDRD3-KD were infected with CVB3 expressing dsRED at an MOI of 0.5. Cells were incubated with virus at indicated time points. Cells from poly(I:C) transfection and CVB3 infection were then lysed and processed for qRT-PCR using qPCR primers specific for indicated targets. Error bars indicate standard deviation. Graphs and error bars processed using GraphPad Prism. Significance was determined using 2-tailed Welch’s T-Test. * = p≤0.05, ** = p≤0.01, *** = p≤0.001.

The results of interferon and ISG expression analyses (Fig 8A) after poly(I:C) transfection reveal a complex association between G3BP1 and TDRD3, individually and in concert, with respect to transcriptional activation and repression of IFNs and ISGs. G3BP1 alone appears to be more important in the activation of IFN-β as well as the ISGs ISG56 (Fig 8A), Viperin, ZAP, and IFI6 (S2C Fig) since depletion leads to significant reductions in transcription of these genes. Interestingly, G3BP1 depletion also limits RNaseL mRNA expression, which is also expressed constitutively and not necessarily an ISG (S2C Fig). TDRD3 appears more important than G3BP1 in the transcriptional activation of IFN-λ1 (Fig 8A) as well as ADAR1 and RTP4 (S2C Fig). Additionally, single knockouts had little effect on IFNλ2/3 expression levels under poly(I:C) stimulation, but expression increased significantly when both G3BP1 and TDRD3 expression was repressed. Conversely, both TDRD3 and G3BP1, individually, play a role in IFNα expression. ISG15 and ISG20 expression may be enhanced by both TDRD3 and G3BP1 additively.

In contrast, TDRD3 may restrict the transcription of IFN-β, ISG56 (Fig 8A), and IFI6 (S2C Fig). G3BP1 appears to repress transcriptional activation of RTP4, a negative regulator of TBK1 and IFN-1 response inhibitor [30] (S2C Fig).

Interestingly, OAS was observed to be transcriptionally repressed by G3BP1 and TDRD3 singly with an additive effect when both G3BP1 and TDRD3 expression are diminished (S2C Fig). These observations suggest dynamic roles played by TDRD3 and G3BP1, alone and together, on the modulation of innate immune signaling during poly(I:C) stimulation.

IFN and ISG transcriptional activation during CVB3 infection resulted in much lower expression fold-changes compared to poly(I:C) stimulation profiles (Fig 8B) in agreement with numerous reports. Enteroviral infection is known to limit expression of ISGs via multiple mechanisms including degradation of IFNAR1 [31] as well as blocking JAK-STAT signaling downstream from the receptors by induction of KPNA1 degradation [32], YTHDF3 cleavage [33], and induction of SOCS1 and SOCS3 expression (negative regulators of JAK-STAT signaling) upon infection [34]. Consistent with enteroviral-mediated JAK-STAT signaling abrogation, we noted no phosphorylated STAT1 in our infected cell lines (S1A Fig). During viral infection, interferon and ISGs expression levels only modestly changed, however KD/KO cells generally exhibited decreased magnitudes of expression of ISG15, IFNλ1, and ISG56 compared to WT cells. We also note a kinetic difference in IFN-β expression between the two cell types, with the KD/KO cells showing a peak of similar magnitude to that observed in WT cells but delayed by 18 hours. We also observed a time-dependent increase in ISG20 expression in KD/KO cells compared to steady expression levels observed across time in WT cells.

**Fig 9:**
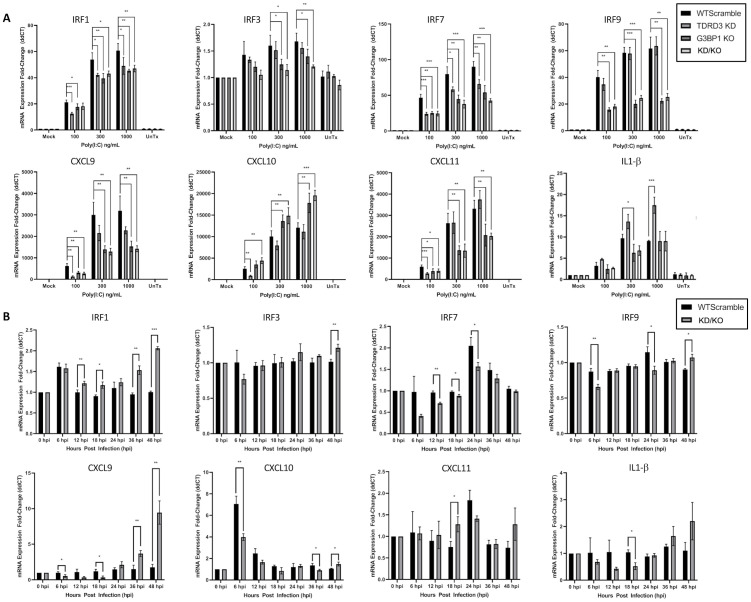
TDRD3 and G3BP1 together modulate transcriptional activation of interferon regulatory factors and pro-inflammatory cytokines. (A) WT HeLa cells possessing scrambled shRNA (WTsc) or G3BP1 knockout HeLa cells possessing scrambled shRNA (G3BP1 KO), TDRD3 knockdown cells, or cells with both G3BP1-KO and TDRD3-KD were transfected with low molecular weight poly(I:C) at concentrations of 0 ng/mL (Mock), 100 ng/mL, 300 ng/mL, 1 μg/mL, or untransfected (UnTx). Cells were incubated for 12 hours post transfection. (B) WT HeLa cells possessing scrambled shRNA (WTsc) or G3BP1 knockout HeLa cells possessing scrambled shRNA (G3BP1 KO), TDRD3 knockdown cells, or cells with both G3BP1-KO and TDRD3-KD were infected with CVB3 expressing dsRED at an MOI of 0.5. Cells were incubated with virus at indicated time points. Cells from poly(I:C) transfection and CVB3 infection were then lysed and processed for qRT-PCR using qPCR primers specific for indicated targets. Error bars indicate standard deviation. Graphs and error bars processed using GraphPad Prism. Significance was determined using 2-tailed Welch’s T-Test. * = p≤0.05, ** = p≤0.01, *** = p≤0.001.

Examination of poly(I:C) stimulation of IRF transcription revealed an additive effect of G3BP1 and TDRD3 on IRF7 expression (Fig 9A). IRF3 also displays this activation pattern but the induction of mRNA expression of this factor was overall very low in HeLa cells. Interestingly, both TDRD3 and G3BP1 may be required to facilitate IRF1 expression, while IRF9 expression appears to be facilitated only by G3BP1. During viral infection (Fig 9B), IRF7 expression was still repressed in KD/KO cells compared to WT cells particularly early on during the infection. In contrast to poly(I:C) transfection, IRF1 expression during infection actually increased over time in the KD/KO cells compared to WT cells. IRF3 and IRF9 expression remained low and generally equivalent between the two cell types during infection.

Considering the interconnectedness of interferon and inflammatory signaling, we assayed for pro-inflammatory cytokines to determine if this aspect of innate immune signaling is modulated by G3BP1 or TDRD3. During poly(I:C) stimulation CXCL9 expression is influenced by both TDRD3 and G3BP1, with stronger transcriptional repression observed in G3BP1 KO cells (Fig 9A). CXCL10 transcriptional activation appeared stronger in G3BP1 KO cells than WT cells, implying that G3BP1 may play some role limiting its activation. This is in contrast to CXCL11 since G3BP1 KO resulted in lower CXCL11 expression, implying that G3BP1 may promote CXCL11 transcription activation. Intriguingly, IL-1β expression increased in TDRD3 knockdown cells but was nearly identical in magnitude to WT cells in G3BP1 KO or KD/KO cells, implying that TDRD3 may play a repressive role in this aspect of inflammation signaling, while G3BP1 may indirectly facilitate this signaling. Conversely, under viral infection (Fig 9B) we found that CXCL9 is increased in the KD/KO cells at later timepoints compared to WT. Additionally, CXCL10 is activated early in infection in WT cells but less so in KD/KO cells. CXCL11 expression during viral infection was generally static in both cell types assayed. Surprisingly, IL-1β expression actually increased in the KD/KO cells at later timepoints, though statistical significance was not observed.

## Discussion

Recently, several effectors of Type I Interferon signaling including PKR, RNaseL, and OAS have been shown to localize to SGs. Our lab has also shown a similar localization to G3BP1-induced SGs, with PKR localizing in a G3BP1 PxxP domain-dependent manner [10]. MDA5 has also been shown to localize to SGs in response to a variety of cell stressors including heat shock, oxidative stress, and viral infection [35]. Similarly, RIG-I has been shown to colocalize to arsenite and virus-induced SGs [9,36]. While not definitive, there is also evidence that MAVS (IPS-1) may localize to SGs while it interacts with PKR, leading to PKR dimerization and activation upon dsRNA binding [37]. Based on these observations it is likely that SGs play a role in IFN-I signaling, and we have hypothesized that virus disruption of cell homeostasis that induces SG formation may concentrate PRRs and promote PRR signaling pathways. Such stress-based PRR signaling may augment a more conventional virus-specific PAMP-PRR activation of innate immunity.

Here we found several additional Type I interferon signaling effectors are recruited to SGs and that there is some selectivity on localization based on the type of stressor. For instance, both IRF3 and TBK1 localized to SGs induced by oxidative stress and G3BP1 over-expression, yet showed a diminished localization to heat- and osmotic stress granules. In contrast, TRAF3 and IRF7 localized to SGs in every stress condition assayed. This indicates that the nature of the stress response may influence the downstream outcome of these signaling pathways. Similarly, MDA5 has been shown to be important for SG formation induced by a mitochondrial stressor even in the absence of colocalization with granules, and its ability to regulate autophagy and SG clearance is dependent upon its role in SG formation [38]. This also supports the role of SG-dependent activation and pathway modulation.

Based on previous reports highlighting the importance of the Tudor domain for localization of TDRD3 to cytoplasmic SGs, we initially hypothesized that TDRD3 localization was driven by the methyl-arginine residues within G3BP1’s RGG domain [19]. Additionally, we previously showed that differential methylation of G3BP1’s RGG domain by PRMTs 1 and 5 along with demethylation by JMJD6 is important for SG kinetics [15,16]. However, our observation that G3BP-null cells treated with arsenite or with overexpression of TDRD3, form TDRD3+ foci indicates that TDRD3’s condensation into SG-like foci is not strictly G3BP1-dependent. Surprisingly, TDRD3 localized to SGs formed by overexpressing the G3BP1 RGG methylation-deficient construct (All-K), which further rules out the idea that TDRD3 is localizing to SGs strictly based upon G3BP1’s methylation status, at least within the RGG domain. A number of scenarios can explain this finding. Firstly, it is possible that TDRD3 may be reading other methylarginine marks on G3BP1, or alternatively, TDRD3 is being recruited by accessory factors rather than directly by G3BP1. It is also possible that TDRD3 itself plays an unknown regulatory role in SG formation or dynamics. Future experimentation using TDRD3 knockout cell lines, as opposed to the TDRD3 knockdown cell lines used in this report, is necessary to determine precise differences in SG formation under stress with or without TDRD3.

Given that TDRD3 overexpression triggers SG formation, and previous work has shown TDRD3 to be a modular protein with scaffolding functions, we hypothesized that TDRD3 may play a major role in the recruitment of innate immune signaling factors to SGs. We first showed that TDRD3 overexpression leads to the formation of SG-like foci possessing many of the same translation initiation factors as canonical SGs even in the absence of G3BP1 and G3BP2. Following this, we assayed these granules and found that the same innate immune signaling factors also localized to these granules as observed in canonical and G3BP1-induced SGs. While more work is required to determine the mechanism(s) involved in the localization of these effectors, our findings highlight the importance of TDRD3 in this recruitment. It is possible that the recruitment of at least IRF3, IRF7, and TBK1 may hinge upon TRAF3 recruitment via TDRD3 interactions. We base this hypothesis on the fact that TDRD3 possesses an ubiquitin-associated (UBA) domain known to bind K63-polyubiquitination marks [39]. TRAF3 along with other TRAF-family proteins, is a K63-specific E3 ubiquitin ligase with auto-ubiquitination activity. We noted that when TDRD3 or G3BP1 expression is repressed, singly or together, TRAF3 recruitment to TIA1 positive SG foci is defective, with no colocalization observed in the double knockout cells. However, further work is needed to determine if TDRD3 is indeed responsible for TRAF3 localization, how this interaction may occur, and subsequent impacts on the localization of the downstream effectors TBK1, IRF3, and IRF7.

Since TDRD3 appears to be an important mediator for the recruitment of IFN-I effectors to SGs and plays a previously unknown role in SG assembly, it presents an attractive target for viral manipulation. Indeed, we show here that TDRD3 itself is cleaved by enteroviral 2A^pro^, similar to 3C^pro^-mediated cleavage of G3BP1 that restricts SG formation over the course of infection [28]. A previous report showed that 2A^pro^ suppresses SG formation and limits IFN-β transcription, yet no SG nucleating components were found to be cleaved by 2A^pro^ [40]. Here, we show that TDRD3 is cleaved by 2A^pro^ and that TDRD3 has an effect on the recruitment of innate immune signaling effectors to SGs as well as the transcriptional activation of many ISGs of the interferon signaling system. Based on these observations, we wanted to explore what impact of the removal of G3BP1 and TDRD3, singly and together, has on enteroviral replication. As presented here, single KO or KD had moderate effects upon enteroviral replication but when both TDRD3 and G3BP1 are knocked out the enteroviral growth rate significantly increases by orders of magnitude as determined by viral genome quantification and infectivity assays. Additionally, there was no apparent difference in enterovirus replication kinetics and host translation shutoff in high MOI infections, indicating potential functional coordination between TDRD3 and G3BP1 at the level of virus infectivity.

Next, we wanted to explore what effects deficiencies in TDRD3 and G3BP1 expression had on interferon pathway components and IFNs themselves, which could help to explain this observation. Overall, the results of the qPCR analyses revealed a complex modulatory association between G3BP1, TDRD3, and the innate IFN signaling pathways during both poly(I:C) stimulation and CVB3 infection. We observed two intriguing findings in WT and KD/KO cells infected by CVB3. Firstly, we noted slower kinetics of IFN-β transcriptional activation in the KD/KO cells compared to WT, with peak expression of IFN-β in WT cells at 6 hours post infection, while KD/KO cells delayed IFN-β production to 18 hours later. Such kinetic delays could be important considering that enteroviruses generally infect, replicate, and release progeny by 6–12 hours post infection [41]. Additionally, KD/KO cells generally showed a decrease in the magnitude of IRF7 transcriptional activation compared to WT cells during the entire course of infection. We also note deficiencies in TRAF3 protein expression during viral infection in the single and double knockouts. These observations suggest that G3BP1 and TDRD3 both play roles in the activation of a robust antiviral state within the cell. Thus, the KD/KO cells show slower kinetics of activation of IFN signaling in triggering an antiviral state. Such hinderance in KD/KO cells would make them more permissible for initial infection and replication at low MOI, in essence increasing the kinetics of enteroviral infection.

We found other interesting patterns of transcriptional activation of IFN types resulting from depletion of G3BP1 or TDRD3 or both together. Of primary interest here is the observation that G3BP1 promoted transcriptional expression of IFN-β (type 1 interferon signaling) while TDRD3 promoted expression of IFNλ-1 (type 3 interferon signaling). Based on these observations it appears that G3BP1 may play a larger role in modulating type 1 interferon signaling, while TDRD3 plays a larger role in modulating the expression of IFN-λ1. To further determine differences between type 1 and type 3 interferon signaling in this context we also assayed SOCS1, which is an inducible negative regulator of IFNλ-induced ISG expression [42,43], and SOCS3, which inhibits IFN-α and IFN-β signaling [44–46]. Based on our findings, and considering the highly tunable nature of interferon signaling, we can conclude that TDRD3 and G3BP1, alone and in tandem, appear to modulate the expression of the JAK-STAT signaling repressors SOCS1 and SOCS3, with G3BP1 playing a larger role in the modulation of SOCS3 signaling.

Alternatively, expression of the transcription factors IRF3, IRF7 and ISG15 and ISG20 appear to be partly promoted by both TDRD3 and G3BP1. Conversely, transcriptional activation of only OAS may be repressed by TDRD3 and G3BP1. Finally, G3BP1 appears to play a dominant role in transcriptional activation of IRF9, ISG56, ZAP, Viperin, RNAse L, and IFI6, whereas TDRD3 may play a role in transcriptional activation of RPT4. The observation that IRF9 mRNA expression is repressed in the G3BP1 knockout and the double G3BP1-KO/TDRD3-KD (KD/KO) cells implies that at least in the case of G3BP1, the effects observed are independent of IFNAR signaling, which was further expanded to TDRD3 KD cells by the presence of phosphorylated STAT1 during poly(I:C) stimulation. Further work is required to determine the mechanism(s) by which G3BP1 and TDRD3 modulate IFN signaling and particularly the distinction between type 1 and type 3 interferon signaling, considering that TDRD3 appears to play a role in activation of IFN-λ1 (type 3 interferon signaling) while G3BP1 appears to more strongly influence IFN-β (type 1 interferon signaling).

One very intriguing observation that emerged in this work is the presence of differential activation of the proinflammatory cytokines and chemokines in WT HeLa cells versus single or double KD/KO cells. During poly(I:C) stimulation, we noted that the inflammatory cytokine IL-1β expression is enhanced in TDRD3 KD only, while G3BP1 KO and KD/KO cells displayed IL-1β expression similar to WT cells. This suggests TDRD3 has a role in repressing IL-1β expression while G3BP1 may indirectly lead to its transcriptional activation. Indeed, previous reports have shown that DDX3X, a SG resident protein required for NLRP3 inflammasome signaling which is involved in IL-1β secretion [47] directly binds to TDRD3’s Tudor domain [19]. Furthermore, considering the patterns of differential expression of type 1 and 3 IFNs, IL-1β, and CXCL9, G3BP1 and TDRD3 may work together in an axis that delineates type 1 and type 3 interferon signaling. Further, TDRD3 may act as a switch between type one interferon signaling through ISR and inflammatory signaling through IFN-λ as well as activation of the NLRP3 inflammasome. Future work will focus on this axis under the context of antiviral SG signaling.

In conclusion, this work expands our understanding of the interplay between the Integrated Stress Response and innate immune signaling. We show localization of several new IFN signaling effectors to stress granules. Moreover, we show that TDRD3 is capable of forming SG-like foci in the absence of the master nucleator G3BP1, and that these granules are also capable of recruiting signaling effectors, and we have found connections between TDRD3 and Type 3 IFN and inflammatory signaling, and G3BP1 to Type 1 IFN expression. G3BP1 and TDRD3 together may help drive the kinetics of the IFN response. This novel role of TDRD3 as a granule nucleator and a putative antiviral protein as well as the idea that it may be working in concert with G3BP1 in this context increases our understanding of its cytoplasmic functions.

## Materials and methods

### Cells, transfections, treatments, and viruses

U2OS and HeLa cells were cultured under standard conditions in DMEM supplemented with 10% BCS. Cell types used were G3BP1/G3BP2 double knockout U20S cells (G3BP-null) or WT U2OS cells, and G3BP1-knockout HeLa cells (G3BP1-KO) or WT HeLa cells as well as WT or G3BP1 KO HeLa cells expressing shRNA against TDRD3 (TDRD3 KD and KD/KO cells respectively). Viral cleavage assays were performed using HEK 293T cells grown under the same conditions. Where applicable, cells were transfected using Lipofectamine 3000 (Thermo Fisher Scientific) according to manufacturer’s instructions using reduced-serum Opti-MEM (Thermo Fisher Scientific) as the transfection media. Oxidative stress: sodium arsenite (Sigma) was added to culture media at 200 μM for 1 hour at 37 °C. Heat shock: cells undergoing heat shock were incubated at 42 °C for one hour. Osmotic stress: D-sorbitol was added to culture media to 500 mM and cells were incubated for one hour at 37 °C. For viral cleavage assays, WT HeLa cells were infected with either PV type 1 (Mahoney Strain), Echo 13, or CVB3-28 at an MOI of 3 in DMEM supplemented with 2% FBS and incubated for 12 hours. For CVB3-induced ISG transcriptional assays, indicated cell types were infected with CVB3 expressing dsRED at an MOI of 0.5. Cells were incubated with virus at indicated time points from 0 to 48 hours. Infectivity assays were performed with indicated viruses as indicated dilutions. Single step growth curves were performed using CVB3 expressing dsRED at an MOI of 3. Poly I:C transfections were performed using low molecular weight Poly I:C (Invitrogen CAS #31852-29-6) using Lipofectamine 3000 without P3000 reagent.

### Plasmid constructs

GFP-G3BP1 methylation site mutant All-K was previously described [15]. Point mutagenesis was performed on G3BP1 All-K using PCR (SuperFi II polymerase Thermo Fisher Scientific) to generate G3BP1 RGG methylation mutants. pSI-TDRD3-eGFP, driven by an SV40 promoter, was adapted from a pEGFP-C1-TDRD3 construct that was generously provided by M. Bedford.

### shRNA knockdown of TDRD3

A pGIPZ construct expressing TDRD3-specific shRNA (OSB Clone ID 200226782 targeting sequence GGGACAATACAGATCATCA) was purchased from the Baylor College of Medicine Cell Based Assay Screening Service (C-BASS) core. WT and G3BP1-KO HeLa T/O cells were transfected with this construct (TDRD3 KD and KD/KO cells, respectively) or a scrambled shRNA control (WTSc or G3BP1KO, respectively) and selected with Puromycin (5 μg/ml) over the course of 3 months until 100% of cells expressed GFP. Knockdown was confirmed via western blot.

### Immunofluorescence (IFA)

Cells were grown on coverslips in 24-well plates. Cells were fixed in 4% paraformaldehyde diluted in PEM buffer (80 mM PIPES pH 6.8, 5 mM EGTA pH 7.0, 2 mM MgCl2) for 15 minutes. Autofluorescence was quenched using sodium borohydrate (1mg/ml) diluted in PEM buffer twice for 5 minutes each followed by permeabilization for 30 minutes (PEM buffer supplemented with 0.5% Triton-X 100). Cells were blocked for one hour at room temperature in 3% BSA in TBS-T (0.1%). Primary antibodies were diluted in blocking buffer. Cells were incubated with primary antibodies at 4°C overnight with gentle rocking. After primary staining, coverslips were washed 3 times with blocking buffer. Cells were incubated with either anti-rabbit or anti-mouse secondary antibodies conjugated to Alexa Fluor dyes (Invitrogen) at 1:500 concentration for 30 minutes at room temperature. Cells were then washed three times in TBS-T followed by PEM buffer. Cells were then fixed again in 4% paraformaldehyde diluted in PEM buffer at RT for 30 minutes. Cells were then washed 3 times in PEM buffer for 5 minutes per wash, quenched twice in sodium borohydrate as previously performed, washed twice in PEM buffer and then washed once in TBS-T. Coverslips were then mounted using Vectashield with DAPI (Vector Laboratories) or Prolong Diamond with DAPI (Invitrogen). Slides were then stored in the dark at 4 °C until imaging. Imaging was conducted on an Olympus IX-71 Deltavision live high resolution deconvolution microscope (GE Healthcare).

### Autoradiography and western blot analysis

Cell pellets from 1–8 x 10^6^ cells were lysed in RIPA buffer and then clarified by centrifugation (10 minutes, 10,000 RPM). Concentrations were determined using a DS-11+ spectrophotometer (DeNovix). Equivalent total protein concentrations were loaded per well and separated in 4–20% gradient tris-glycine polyacrylamide gels (Invitrogen) and transferred onto nitrocellulose membranes (Whatman). Blots were then blocked in Blotto (5% nonfat dry milk diluted in TBS-T) for 1 hour at RT. Blots were then incubated with primary antibodies at specified concentrations listed in Table 1. Licor red and far red secondary antibodies were used at a concentration of 1:20,000. Western blot imaging was performed on a Licor CLx system. For autoradiography analysis, cells were radiolabeled with ^35^S-Met/Cys (EXPRESS Protein Labeling Reagent, Perkin Elmer) with 25 μCi/ml for 60 min. Cells were lysed in RIPA buffer and then resolved on 10% polyacrylamide gels and dried. Dried gels were exposed to film (Kodak).

**Table 1:** Antibodies.

Antibody	Host	Manufacturer	Identification	Concentration
IRF3 (IFA)	Rabbit	Cell Sig. Tech.	11904S	1:250
IRF7 (IFA)	Rabbit	Abcam	ab109255	1:250
TBK1 (IFA)	Rabbit	Invitrogen	711315	1:250
TRAF3 (IFA)	Rabbit	Biorbyt	orb214703	1:500
TRAF3 (WB)	Rabbit	Proteintech	18099-1-AP	1:750
TDRD3 (IFA)	Rabbit	Invitrogen	PA5-84749	1:500
TDRD3 (WB)	Rabbit	Cell Sig. Tech.	5942S	1:1000
G3BP1 (IFA)	Rabbit	Made In-House	NTD	1:1000
Tia1 (IFA)	Mouse	Santa Cruz	sc-166247	1:250
STING (IFA)	Mouse	Novus	MAB7169-SP	1:500
cGAS (IFA)	Rabbit	Novus	NBP1-86761	1:500
p-STAT1 (Tyr701)	Rabbit	Invitrogen	700349	1:500
puromycin	Rabbit	Millipore	MABE343	1:500

### Immunofluorescence analysis and quantifying colocalization index (C.I.)

Cells were plated as described for immunofluorescence, transfected with GFP-tagged WT (TDRD3 or G3BP1) or G3BP1-RGG mutants as described for transfection, fixed, and then stained for indicated antigens. Stress granules were then defined by processing images from the GFP channel (488 nm) such that only granules were assigned a value of 255 and background was assigned a value of 1 making stress granule mask images. Images from stained channels (594 nm and 647 nm) as well as the stress granule mask images were processed using Cell Profiler [48,49]. Briefly, a Cell Profiler analysis pipeline was created that defined granules from the stress granule mask image as primary objects ranging from 10 to 500 pixels. Secondary objects demarcating cytoplasmic staining regions for analysis were defined as the region 10 pixels around granules with no overlap on other granules or nuclei. Tertiary objects were defined as the area between the periphery of stress granules and the periphery of the secondary objects. Average intensity within the stress granule regions as well as that within the tertiary region surrounding stress granules (Arbitrary Intensity Units) were then calculated for each stress granule per image with an average of 7 images per transfected construct encompassing a range of 100 to 400 granules assayed per transfection. A ratio between the staining intensity within the stress granules and around the stress granules was then calculated for each granule. Average intensities per group were then calculated and plotted using Graphpad Prism (version 9).

### RNA extraction and RT-qPCR

qPCR analysis was performed in quadruplicate with technical duplicates using primers for targets listed in Table 2. After specified treatments, cells grown in 24-well culture plates were washed thrice in ice-cold PBS and harvested with TRIzol reagent (Ambion). Samples were homogenized by pipetting up and down five times per sample. Samples were incubated for 5 minutes at room temperature. Chloroform was added to samples and incubated for an additional 3 minutes at room temperature. Samples were centrifuged for 15 minutes at 12,000 x g at room temperature. Aqueous layer was retained. RNA was precipitated by incubating with isopropanol at -20°C for 20 minutes. Samples were then centrifuged for 10 minutes at 12,000 x g at room temperature. RNA pellets were retained. Pellets were washed in 90% ethanol and centrifuged for 5 minutes at 8,000 x g. Supernatant was discarded and pellets were air dried at room temperature for 5–10 minutes. RNA was resuspended in 40 μl of RNase-free HyClone HyPure water (Cytiva). DNase treatment of RNA was performed using Turbo DNA-free kit (Invitrogen) per manufacturer instructions. cDNA synthesis was performed using Protoscript II RT (NEB) with random hexamers (NEB) per manufacturer protocol. cDNA amplification was performed using Power SYBR-Green mastermix (Thermo Fisher Scientific) on a QuantStudio 3 RT-PCR system (Thermo Fisher Scientific). Quantification was performed using ddCT method for mRNA expression analysis or absolute quantification of viral genomes were determined using standard curve analysis.

**Table 2:** qPCR Primers.

Target	Forward (5’ - 3’)	Reverse (5’ - 3’)
huIFNα	GACTCCATCTTGGCTGTGA	TGATTTCTGCTCTGACAACCT
huIFNβ	AAGGCCAAGGAGTACAGTC	ATCTTCAGTTTVGGAGGTAA
huIFNƛ1	CGCCTTGGAAGAGTCACTCA	GAAGCCTCAGGTCCCAATTC
huIFNƛ2/3	AGTTCCGGGCCTGTATCCAG	GAGCCGGTACAGCCAATGGT
huSOCS1	TTCGCCCTTAGCGTGAAGATGG	TAGTGCTCCAGCAGCTCGAAGA
huSOCS3	CATCTCTGTCGGAAGACCGTCA	GCATCGTACTGGTCCAGGAACT
huCXCL9	CTGTTCCTGCATCAGCACCAAC	TGAACTCCATTCTTCAGTGTAGCA
huCXCL10	GGTGAGAAGAGATGTCTGAATCC	GTCCATCCTTGGAAGCACTGCA
huCXCL11	AAGGACAACGATGCCTAAATCCC	CAGATGCCCTTTTCCAGGACTTC
huIL-1β	AAACAGATGAAGTGCTCCTTCCAGG	TGGAGAACACCACTTGTTGCTCCA
huIRF1	GAGGAGGTGAAAGACCAGAGCA	TAGCATCTCGGCTGGACTTCGA
huIRF3	TCTGCCCTCAACCGCAAAGAAG	TACTGCCTCCACCATTGGTGTC
huIRF7	AGGGTGACAGGTACGGCTCT	CTCCTGGAGAGGGACAAGAA
huIRF9	CCACCGAAGTTCCAGGTAACAC	AGTCTGCTCCAGCAAGTATCGG
huISG20	TGGACTGCGAGATGGTGG	GGGTTCTGTAATCGGTGAT
huOAS1	GCCCTGGGTCAGTTGACTGG	TGAAGCAGGTGGAGAACTCGC
huViperin	CCAGTGCAACTACAAATGCGGC	CGGTCTTGAAGAAATGGCTCTCC
huZAP	GATGATGCTGACCCAAGAGTAGC	GGACAACCCTTACACAGATGGTC
huISG15	GAGCTAGAGCCTGCAGCAAT	TTCTGGGCAATCTGCTTCTT
huRNaseL	AAGGCTGTTCAAGAACTACACTTG	TGGATCTCCAGCCCACTTGATG
huISG56/IFIT1	CAGCAACCATGAGTACAAAT	AAGTGACATCTCAATTGCTC
huIFI6	TGATGAGCTGGTCTGCGATCCT	GTAGCCCATCAGGGCACCAATA
huRTP4	GACGCTGAAGTTGGATGGCAAC	GTGGCACAGAATCTGCACTTGG
huADAR1	CATGGCTTTGCTGCTGAAT	CTGCTTGCCTTGCTTCTTG
huRPS18	TGTGGTGTTGAGGAAAGCA	CTTCAGTCGCTCCAGGTCTT
Enterovirus	CGGCCCCTGAATGCGGCTAA	GAAACACGGACACCCAAAGTA

## Supporting information

S1 Fig (A) JAK-STAT signaling occurs in WT HeLa, TDRD3 KD, G3BP1 KO, and KD/KO cells during poly(I:C) transfection but not CVB3 infection. Cells were infected with CVB3 (MOI 0.5) and incubated for 24 hours or transfected with 300 ng/mL LMW poly(I:C) and incubated for 12 hours. Cells were harvested and lysed in RIPA buffer supplemented with phosphatase and protease inhibitors (HALT cocktail). 100 ug whole cell lysate was analyzed via SDS-PAGE followed by western blotting for phosphorylated STAT1. (B) 100 ug whole cell lysate was analyzed via western blot for TRAF3 followed by densiometric quantification relative to gapdh. (C) TDRD3 overexpression shuts down host translation with or without G3BP1. WT or G3BP KO U2OS cells were grown on cover slips and then transfected with a GFP-tagged TDRD3 or G3BP1 expression construct. Cells were pulsed with 50 ug/mL puromycin for 10 minutes. Immunofluorescence was performed using an antibody against puromycin (Millipore MABE343). (D) Loss of TDRD3, G3BP1, or both TDRD3 and G3BP1 results in hindered SG formation and diminished TRAF3 localization during osmotic stress. Cells were grown on cover slips and treated with sodium arsenite for 1 hour. IFA was performed using antibodies against Tia1 (green, SG marker) and TRAF3 (red).
(TIF)

S2 Fig qPCR expression analysis of SOCS1, SOCS3, and additional ISGs. (A) qPCR analysis of SOCS1 and SOCS3 expression was performed in WT HeLa, TDRD3 KD, G3BP1 KO, and KD/KO cells transfected with poly(I:C) at indicated concentrations and incubated for 12 hours or (B) infected with CVB3 for and incubated with virus for indicated time points. (C) qPCR analysis of additional ISG expression in poly(I:C) transfected cells.
(TIF)
