# Supplementary figures and images for: TDRD3 is an antiviral restriction factor that promotes IFN signaling with G3BP1
Source: PLoS Pathog. 2022 Jan 27;18(1):e1010249. doi: 10.1371/journal.ppat.1010249 (PMC8824378; doi:10.1371/journal.ppat.1010249)

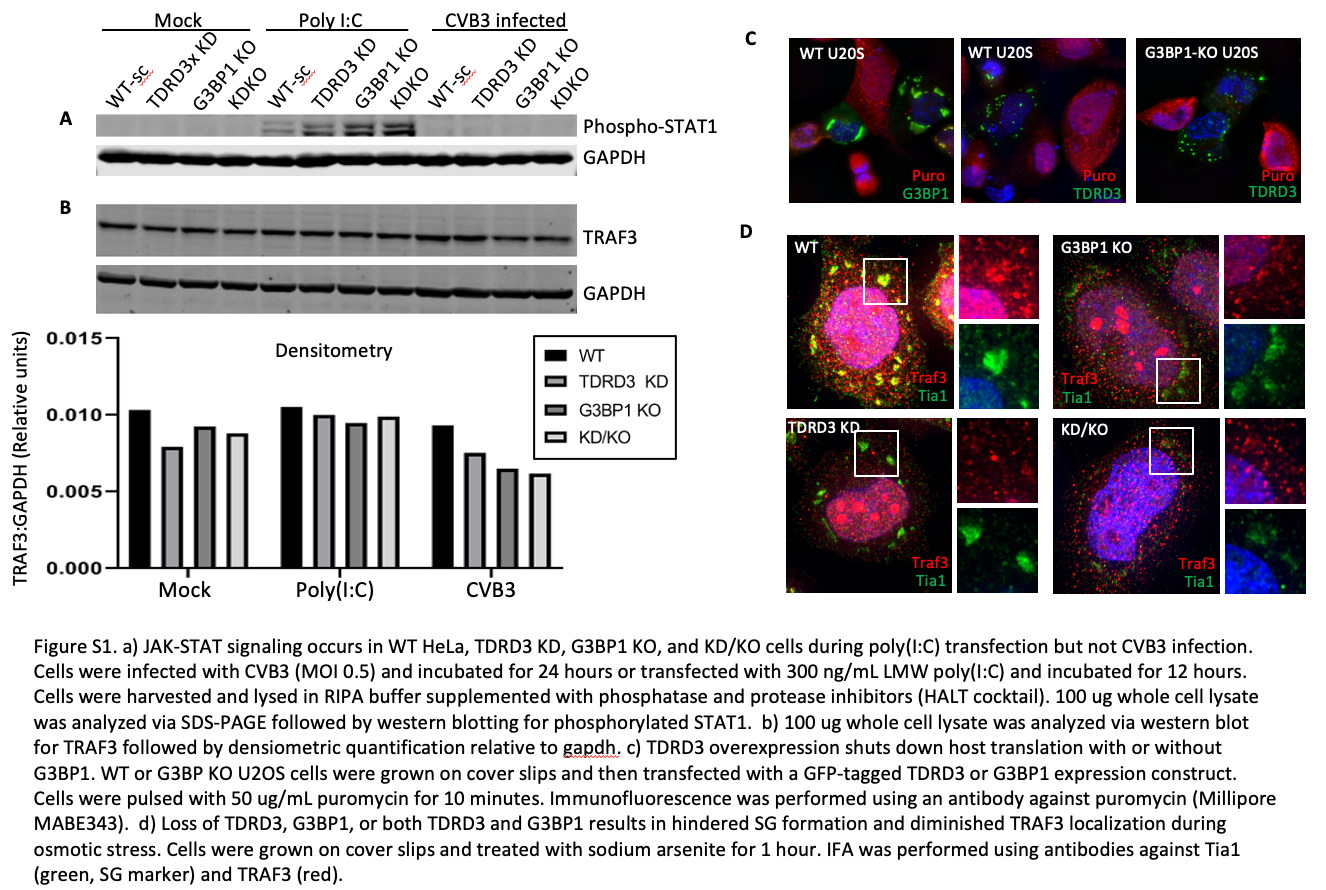

Supplement: S1 Fig — (A) JAK-STAT signaling occurs in WT HeLa, TDRD3 KD, G3BP1 KO, and KD/KO cells during poly(I:C) transfection but not CVB3 infection. Cells were infected with CVB3 (MOI 0.5) and incubated for 24 hours or transfected with 300 ng/mL LMW poly(I:C) and incubated for 12 hours. Cells were harvested and lysed in RIPA buffer supplemented with phosphatase and protease inhibitors (HALT cocktail). 100 ug whole cell lysate was analyzed via SDS-PAGE followed by western blotting for phosphorylated STAT1. (B) 100 ug whole cell lysate was analyzed via western blot for TRAF3 followed by densiometric quantification relative to gapdh. (C) TDRD3 overexpression shuts down host translation with or without G3BP1. WT or G3BP KO U2OS cells were grown on cover slips and then transfected with a GFP-tagged TDRD3 or G3BP1 expression construct. Cells were pulsed with 50 ug/mL puromycin for 10 minutes. Immunofluorescence was performed using an antibody against puromycin (Millipore MABE343). (D) Loss of TDRD3, G3BP1, or both TDRD3 and G3BP1 results in hindered SG formation and diminished TRAF3 localization during osmotic stress. Cells were grown on cover slips and treated with sodium arsenite for 1 hour. IFA was performed using antibodies against Tia1 (green, SG marker) and TRAF3 (red). (TIF) [file ppat.1010249.s001.tif]

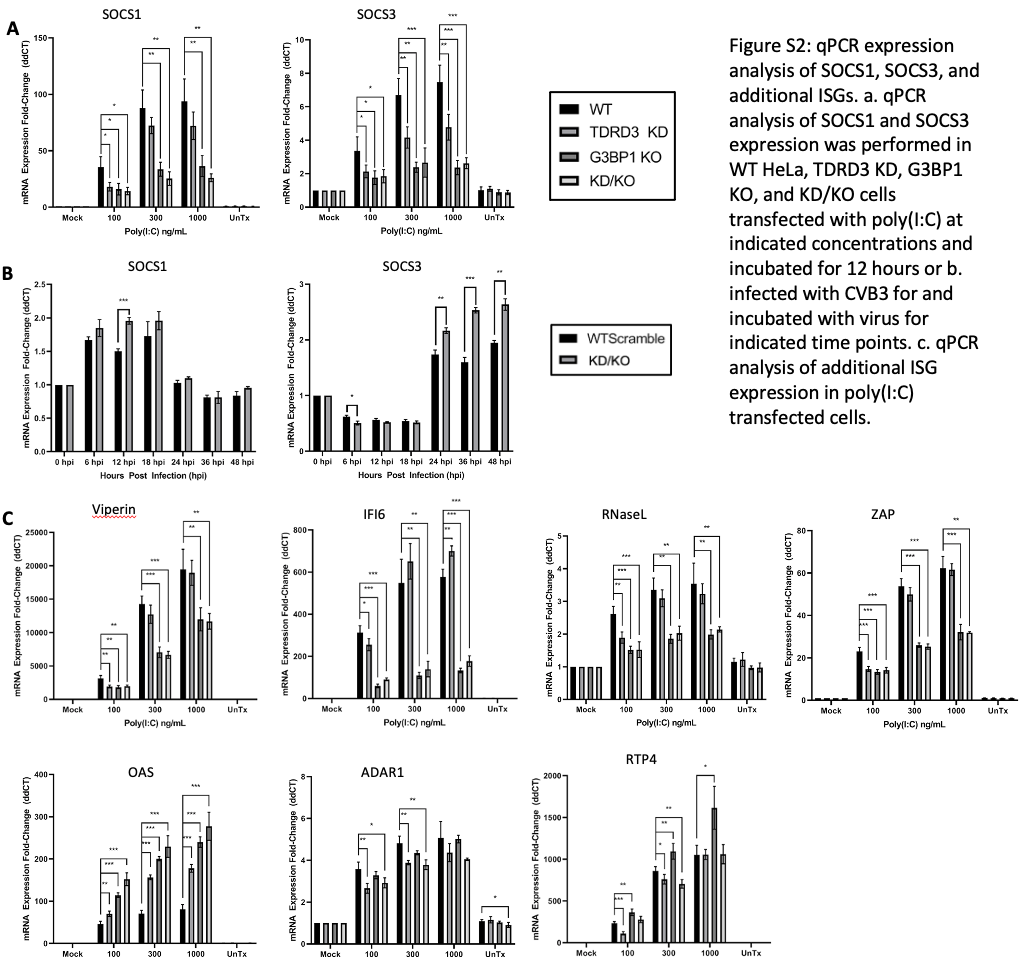

Supplement: S2 Fig — (A) qPCR analysis of SOCS1 and SOCS3 expression was performed in WT HeLa, TDRD3 KD, G3BP1 KO, and KD/KO cells transfected with poly(I:C) at indicated concentrations and incubated for 12 hours or (B) infected with CVB3 for and incubated with virus for indicated time points. (C) qPCR analysis of additional ISG expression in poly(I:C) transfected cells. (TIF) [file ppat.1010249.s002.tif]
